# Total Phenolic and Yellow Pigment Contents and Antioxidant Activities of Durum Wheat Milling Fractions

**DOI:** 10.3390/antiox6040078

**Published:** 2017-10-14

**Authors:** Bin Xiao Fu, Constance Chiremba, Curtis J. Pozniak, Kun Wang, Shin Nam

**Affiliations:** 1 Grain Research Laboratory, Canadian Grain Commission, 1404-303 Main Street, Winnipeg, MB R3C 3G8, Canada; Constance.Chiremba@grainscanada.gc.ca (C.C.); kun.wang@grainscanada.gc.ca (K.W.); shin.nam@grainscanada.gc.ca (S.N.); 2 Crop Development Centre, University of Saskatchewan, 51 Campus Drive, Saskatoon, SK S7N 5A8, Canada; curtis.pozniak@usask.ca

**Keywords:** durum wheat, milling fractions, carotenoid and phenolic content, antioxidant activity

## Abstract

The aim of this study was to investigate the distribution of total yellow pigments, total phenolic compounds, and their antioxidant activities in various durum wheat milling fractions. Carotenoid composition of yellow pigment extract was also examined using UPLC. The ABTS radical scavenging activity of the milling fractions decreased in the order of short bran/bran > feed flour > flour/semolina in both total phenolic and total yellow pigment extracts. Yellow pigments extracts from bran, short bran, and feed flour exhibited 5.6–15.4% higher antioxidant activity than those of total phenolic extracts from the corresponding milling fractions. The UPLC results showed a non-carotenoid peak at Rt 0.47 min which was present in fractions of the grain outer layers but absent in semolina and flour. This peak absorbed in the UV range of 271 to 327 nm. These observations suggest that the unknown peak could be composed of phenolic compounds co-extracted in their free form with carotenoids in the polar water-saturated butanol solvent. The compounds in this peak could result in overestimation of carotenoid content and antioxidant activity in bran, short bran and feed flour as the peak contributed to 18.3–26.0% of total carotenoids if it was taken into account.

## 1. Introduction

The beneficial effects of whole wheat consumption are due to their high and peculiar content in bioactive phytochemicals, which include a wide variety of compounds: phenolics, carotenoids, flavonoids, anthocyanidins, lignans, tocotrienols, and phytosterols [1]. These plant derived secondary metabolites are high in antioxidant activity and function as reducing agents, radical scavengers, and metal ion chelators among others [2,3]. Carotenoids are of particular interest for durum wheat because they are responsible for the yellow color of semolina and end products such as pasta and couscous. Phenolic acids are synthesized as part of multifunctional defense system against biotic and abiotic stresses in plants. Over 80% of total phenolic acids in wheat are bound to cell wall polysaccharides [4,5]. While phenolic acids act as antioxidants by donating hydrogen or electrons, carotenoids contribute to antioxidant activity by quenching singlet oxygen and free radicals. The ability of carotenoids to function as antioxidants is influenced by the presence of functional groups with increasing polarity such as carbonyl and hydroxyl groups in the terminal ring and the number of conjugated bonds [6]. There were significant variation of total antioxidant activity, total phenolics and yellow pigments in durum wheat as function of genotype, environment, and their interactions [7].

Phenolic compounds and carotenoids are unevenly distributed across the grain kernel, hence their concentrations differ significantly in milling streams or kernel fractions [8,9]. For all cereal grains, the germ and aleurone have significantly higher levels of carotenoids than the starchy endosperm. The antioxidant activity of wheat aleurone layer was found approximately a third to half of the antioxidant activity observed in bran fractions [9]. A study on the effect of milling on antioxidant potential showed total phenolic content (TPC) and antioxidant activity are concentrated in the outermost layers, thus the bran fraction alone demonstrated a higher antioxidant activity than other milling fractions [8]. Žilić et al. (2012) reported that approximately 99% of total phenolic compounds in durum wheat were attributed to bran and only 1% to flour [10]. However, the contribution of flour to total antioxidant activity was 29%, suggesting that carotenoids were the most potent antioxidants in durum wheat flour since only trace amount of phenolic compounds were present. Carotenoids are the most abundant antioxidant compounds in wheat endosperm.

Carotenoids are usually estimated by quantifying total yellow pigments based on spectrophotometric determination. Extraction of yellow pigments with organic solvents, such as water saturated butanol (WSB), is not specific. Other free and soluble compounds can also be extracted along with carotenoids. Use of colorimetric methods may overestimate the quantity of carotenoids. Levizou et al. (2004) showed that phenolic acids were co-extracted with carotenoids in crude twig extracts [11]. The authors suggested that absorbance of the co-extracted phenolic acids extends into the visible range interfering with carotenoid estimation, and consequently overestimating yellow pigment content. According to Fratianni et al. (2005), extraction of whole meal with WSB resulted in overestimation of total carotenoid content due to interfering co-extracted pigments from the seed coat [12]. Some overestimation (~20%) of the colorimetric method in comparison to HPLC was noticed by Abdel-Aal et al. (2007) [13]. Brandolini et al. (2008) concluded that although carotenoids are the most important pigments in determining the yellow color of wheat flour, a definitive and precise measurement of carotenoids can be achieved only by HPLC analysis [14]. Literature reported varying levels of carotenoid contribution to total yellow pigment (TYP) content in durum wheat. Hentschel et al. (2002) reported a major contribution (50%) of unknown substances to the yellow color of semolina [15]. By comparing total carotenoid concentration as determined by HPLC with TYP of WSB extract, Digesù et al. (2009) showed that the portion of carotenoids amounted to 33.2% of the yellow pigments in the 80 cultivated and wild tetraploid wheats [16]. Blanco et al. (2011) demonstrated that total carotenoids concentration amounted to 37% of the yellow pigments, indicating unknown color-producing compounds in the durum extracts [17]. However, there have been no studies that identify and characterize these unknown compounds in durum wheat.

The main objective of this study was to evaluate the contents of total yellow pigments, total phenolic compounds, and their antioxidant activities in milling fractions of two durum wheat genotypes with and without *Lpx-B1.1* gene deletion. The composition and quantity of carotenoids in different milling fractions was analyzed using UPLC. In view of the suggestions that compounds such as phenolic acids can co-extract with carotenoids, this study further characterized spectra of eluted compounds to determine the presence and nature of interfering compounds.

## 2. Materials and Methods

### 2.1. Durum Wheat Samples and Milling

Two popular Canadian durum cultivars, Strongfield (without *Lpx-B1.1* gene deletion) and Navigator (with *Lpx-B1.1* gene deletion), were used for this study. Both cultivars are checks for the Canadian Durum Wheat Variety Registration Trials. Duplicate samples (1.5 kg) of Strongfield and Navigator were milled on a four stand Allis-Chalmers laboratory mill coupled with a laboratory purifier according to the mill flow described by Dexter et al. (1990) [18]. Samples were tempered to 16% moisture before milling. The mill room was controlled at 21 °C and 60% R.H. The milling process consists of four corrugated break roll passages, five corrugated sizing roll passages, and 10 purification steps. Roll gaps on the first three breaks are kept relatively wide (1.29 mm, 0.41 mm, and 0.20 mm) to produce semolina with coarse granulation. After the break passages, particles retained on 425 μm screen were collected as bran. The coarser material retained on a 630 μm screen was passed through the sizing rolls and sifted on a box sifter. After sizing passages, streams with fine particles passing through the 180 μm sieve were collected as flour, whereas particles retained on a 700 μm were collected as shorts. The remaining streams were passed through the purifier. Semolina streams were collected after each purification step. Streams with particles passing through the 571 μm screens but retained on the 183 μm screens were collected as semolina. Particles retained on the 630 μm screens on purifiers 7 to 10 were collected as feeds. The milling yields for various fractions were 12% bran, 6% short bran, 4% feed flour, 69% semolina and 8% flour on average. The obtained materials from various milling fractions were kept at 4 °C before analyses.

### 2.2. Total Yellow Pigment Extraction and Measurement

The micro-procedure for measuring total yellow pigment (TYP) was followed [19]. Samples of milling fractions (200 mg) were extracted with 1 mL water-saturated butanol (WSB) in 2 mL capacity micro-centrifuge tubes. The mixture was homogenized at 30 Hz for 5 min using a TissueLyser II bead mill (Qiagen, Hilden, Germany), then rested for 1 h at room temperature, and vortexed for 15 s before centrifugation for 10 min at 15,000× *g*. Absorbance of supernatants was measured at 449 nm using a UV/Visible spectrophotometer in triplicate. Total yellow pigment content was calculated by averaging the absorbance of supernatants of each sample at 449 nm and converting to yellow pigments according to the absorption coefficient of lutein (A^1%^ = 2474) [20]. TYP (μg/g) = (86/(100-percent moisture)) × ((Abs1 + Abs2 + Abs3)/3) × 21.4 (14% m.b.).

### 2.3. Analysis of Total Phenolic Content

A modified Folin-Ciocalteu method [21] was used to measure total phenolic content in the milling fractions. Samples (200 mg) of whole meal, bran, short bran, feed flour, semolina and flour were extracted with 1.0 mL acidified methanol (1% concentrated HCl in methanol, *v*/*v*). The sample and solvent mixtures containing 5.73 mm stainless ceramic beads were homogenized at 30 Hz for 5 min using a TissueLyser II bead mill (Qiagen, Hilden, Germany) and rested at room temperature for 2 h before centrifugation. The supernatants were reacted with 7.5 mL Folin Ciocalteu phenol reagent and 2.5 mL sodium carbonate (20%, *w*/*v*) for 2 h. Absorbance was read at 760 nm. Total phenolic acid content was also measured in yellow pigment samples extracted with water saturated butanol. Catechin was used as a standard.

### 2.4. Trolox Equivalent Antioxidant Capacity Assay

Antioxidant activity of both yellow pigment and phenolic extracts was determined using the ABTS radical scavenging assay according to Awika et al. (2003) [22]. In a typical experiment, 2,2’-azinobis (3-ethylbenzothiazoline-6-sulfonic acid) (ABTS) radical solution was generated via interaction of 3 mM of K_2_S_2_O_8_ with 8 mM of ABTS salt in deionized water for 16 h in the dark. This was followed by diluting ABTS radical solution with phosphate buffer solution (Ph 7.4) in 150 mM NaCl to obtain an initial absorbance of 1.5 at 734 nm. Antioxidant activity was measured by adding 100 μL of the extract or standard (Trolox) directly into 2900 μL of ABTS radical solution. Absorbance was read at 734 nm after reaction of ABTS radical with carotenoid extracts or standard for 30 min.

### 2.5. UPLC Analysis of Carotenoids

Analysis of carotenoids of total yellow pigment extracts of milling fractions were carried out using an Acquity H Class UPLC system (Waters, Milford, MA, USA) coupled with C18 BEH 300 column according to Hung and Hatcher (2011) with some modifications [23]. The column and sample temperatures were set at 35 °C and 25 °C, respectively. Extracts were filtered through a 0.2 μm Polyvinylidene Difluoride (PVDF) membrane, and injection volume was 3 μL. The mobile phase consisted of a quaternary solvent system, A—Millipore water, B—methanol, C—isopropanol, and D—acetonitrile. The flow rate was controlled at 0.6 mL/min. The gradient elution profile, which lasted 6 min, was as follows: 15% A to 5% A in 1 min, 1% A to 1% A in 3 min, 15% A to 15 A in 2 min. The peaks were detected at 449 nm with a Waters photodiode array detector. Carotenoid composition and content were determined using peak absorption maxima and the area under the detected peaks, respectively. Carotenoids were identified by their elution sequence and UV/Visible absorption spectra. Pigment identification was based on the method by Hung and Hatcher (2011), who separated carotenoids in durum wheat using the same UPLC equipment and column as in this study [23].

### 2.6. Statistical Analyses

Durum wheat samples were milled in duplicate. Total yellow pigment content, total phenolic content, and antioxidant activity assays on the milling fractions were measured in triplicate. All data analyses were performed using SPSS version 9.0 (SPSS, Inc., Chicago IL, USA). Tukey’s test following one way ANOVA indicated significant different with a level of *p* < 0.05. Results are expressed on 13.5% and 14.0% moisture basis for whole meal and all milling fractions, respectively.

## 3. Results and Discussion

### 3.1. TYP Content of Durum Wheat Milling Fractions

TYP content is an important parameter in the evaluation of advanced durum wheat breeding lines for registration in Canada. Results showed that TYP content varied among the milling fractions of both genotypes examined (Table 1), indicating yellow pigments were unevenly distributed along durum wheat kernels. The TYP content of the milling fractions ranged from 7.40–8.65 μg/g and 7.83–8.94 μg/g for Navigator and Strongfield, respectively. It is interesting to note that the yellow pigment concentration as determined by the spectrophotometric method increased significantly from the bran toward the endosperm for Navigator. In contrast, Strongfield shows an opposite distribution pattern: the yellow pigment concentration decreased from the outer layers to the endosperm. As check varieties in the Canadian durum wheat variety registration trials, Navigator has consistently shown higher semolina TYP content (by ~1 μg/g) than Strongfield over the years, although there was little difference in pigment content of whole wheat meal between the two cultivars (results not shown). These results indicate that pigments contributing to the yellow color are unevenly distributed in the wheat kernel, and the distribution pattern is dependent on genotype. This is in agreement with results of Borrelli et al. (1999), who demonstrated that pigment loss from wheat to semolina varied with genotypes [24]. However, comparing the amounts of color-related components in whole meal and semolina, Borrelli et al. (2008) noted that milling process did not substantially affect the distribution of yellow pigments [25]. Results obtained by Hentschel et al. (2002) showed higher levels of TYP in outer layers of durum wheat grain than the inner layers [15]. Results of Panfili et al. (2004) showed that lutein was equally distributed along the wheat kernel [26], while Ndolo and Beta (2013) found that lutein is concentrated in the germ [9].

### 3.2. Total Phenolic Content and Antioxidant Activity of Acidified Methanol Extracts

Total phenolic content (TPC) of various milling fractions are shown in Table 1. The TPC of different fractions ranged from 32.1 to 101.2 mg catechin equivalents (CE)/100 g and from 33.1 to 116.1 mg CE/100 g for Navigator and Strongfield, respectively. In both cultivars, the short bran fraction had the highest TPC. Phenolic content of the milling fractions decreased in the order of short bran > bran > feed flour > flour and semolina. The concentration of total phenolics in the semolina and flour fractions was approximately one third of that in bran and short bran. These results are in agreement with findings of Beta et al. (2005), who examined the distribution of phenolics and antioxidant activities in common wheat fractions derived from pearling and roller milling [27].

**Figure 1:**
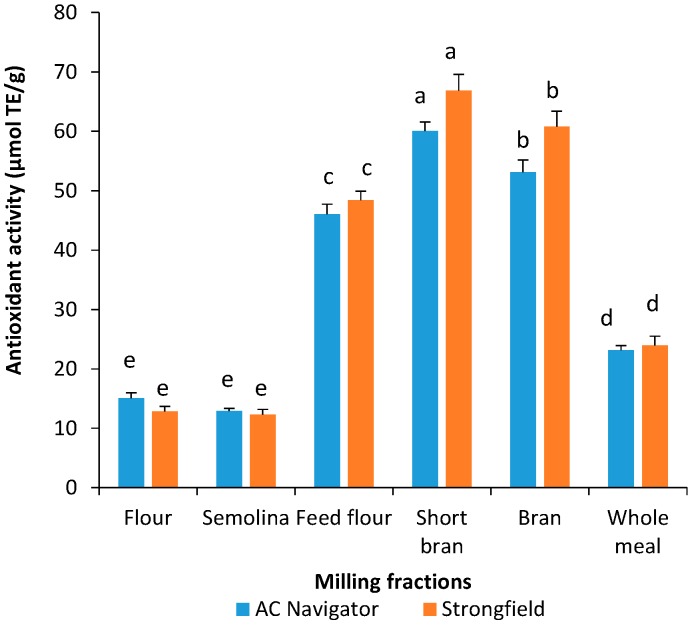
Free radical scavenging activity of total phenolic extracts using acidified methanol on 2,2’-azinobis (3-ethylbenzothiazoline-6-sulfonic acid) (ABTS) radical. Vertical bars represent standard deviations. Different letters indicate significant differences at *p* < 0.05 for each genotype.

The antioxidant activities of durum wheat milling fractions varied significantly (Figure 1a), ranging from 13 to 61 μmoL TE/g for Navigator and 12 to 68 μmoL TE/g for Strongfield. Phenolics are effective quenchers of the ABTS radicals. High total phenolic content as observed in the grain outer layers (i.e., bran, short bran and feed flour) (Table 1) could correspondent to the high antioxidant activity in the milling fractions (Figure 1a), while fractions from the endosperm (i.e., semolina and flour) had lower antioxidant activity and phenolic content. Liyana-Pathirana and Shahidi (2007) reported phenolic content and ABTS radical scavenging activity decreased in the order of bran > shorts > feed flour > whole meal > flour for durum wheat milling fractions after defatting [8]. Zhou et al. (2004) investigated the antioxidant properties of whole grain, bran, and aleurone layer of a Swiss red wheat variety [28]. Compared to whole grain and bran, they concluded that aleurone layer exhibited the highest concentration of phenolic acids and thus radical scavenging activity. Accordingly, the short bran fraction, which is known to be rich in aleurone and germ, could contribute to its highest antioxidant activity in comparison to other milling fractions. Furthermore, ferulic acid, which occurs in high concentration in wheat aleurone layer and is cross-linked with arabinoxylans (Fulcher et al., 1972), could be responsible for the high antioxidant activity observed [29]. Whole meal had approximately 50% the antioxidant activity of short bran. The lower phenolic content and antioxidant activity in whole wheat is simply due to the dilution of the phenolic compounds by the starch-rich endosperm.

### 3.3. Total Phenolic Content and Antioxidant Activity of Water-Saturated Butanol Extracts

This study also sought to validate the assumption that phenolic compounds could be co-extracted with carotenoids as suspected by Levizou et al. (2004) [11]. It is clear that total phenolic content in yellow pigment extracts with WSB ranged from 17.3–73.3 mg CE/100 g among the milling fractions (Table 1). For the same milling fraction, the phenolic content in yellow pigment extract was 35–40% lower than that of phenolic extract with acidified methanol. As expected, phenolic compounds in pigment extracts were more concentrated in the grain outer layers than the endosperm, as indicated by much higher TPC in bran fractions. Lavelli et al. (2009) quantified the soluble phenolic compounds in WSB extracts of whole meal samples and found out the gallic acid equivalents of WSB extracts ranged from 329 to 400 mg/kg for durum wheat species [30]. It appears that phenolic compounds could be extracted simultaneously with carotenoids in the polar water-saturated butanol solvent, such that the determination of total carotenoids using spectrophotometric methods could be overestimated. The overestimation of carotenoids has been widely recognized [9,12,14,15,16,17] and was mostly attributed to unknown compounds in extracts for yellow pigments. Abdel-Aal et al. (2007) reported that the colorimetric method of total yellow pigments overestimated chromatography by about 23% for lutein and 20% for total carotenoids [13]. Fratianni et al. (2005) suggested that the colorimetric measurement of total carotenoids from yellow pigment extracts may be inapplicable to whole meal, as total carotenoid content could be overestimated by interfering pigments found in bran [12]. Yellow pigment should be considered useful only for an approximate appraisal of total carotenoids content. A definitive and precise measurement of carotenoids can be achieved by HPLC analysis.

Yellow pigment extracts of the milling fractions were also evaluated for their free radical scavenging activity against the ABTS^•+^ radical cation. The major carotenoids reported in wheat are lutein and zeaxathin and their ability to quench the ABTS^•+^ radical cation is modulated by the presence of a hydroxyl group at each of the β-rings [6]. The fraction with highest ABTS^•+^ scavenging capacity was the short bran, followed by bran, feed flour, whole meal and lastly semolina and flour (Figure 1b). For semolina and flour, the pigment extracts with WSB had lower antioxidant activity (6.2–8.4 μmoL TE/g) than that of phenolic extracts with acidified methanol (10.8–13.0 μmoL TE/g). Ndolo and Beta (2013) reported that carotenoid content and DPPH scavenging activity in manually dissected wheat fractions were in order of germ > aleurone > endosperm [9]. A direct comparison of results between research groups is difficult because of the variation in materials, extracting solvents, and measuring systems.

**Figure 2:**
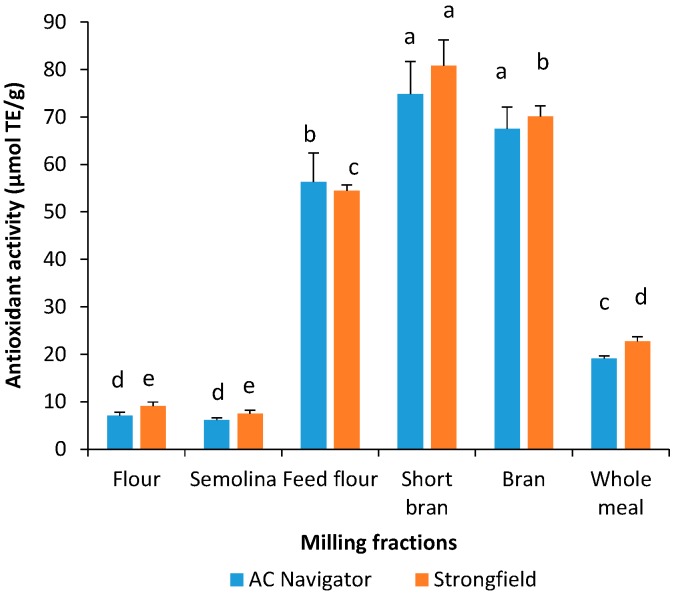
Free radical scavenging activity of total yellow pigment extracts using water-saturated butanol on ABTS radical. Vertical bars represent standard deviations. Different letters indicate significant differences at *p* < 0.05 for each genotype.

It is of interest to note that outer layer fractions (bran, short bran, and feed flour) of durum wheat had higher antioxidant activity measured in yellow pigment extracts than extracts using acidified methanol (Figure 1 and Figure 2). The ABTS^•+^ scavenging capacity of yellow pigment extracts in bran, short bran and feed flour was 5.6–15.4% higher than the antioxidant activity in phenolic extracts with acidified methanol. The results indicate that in addition to carotenoids, the co-extracted phenolic compounds in the yellow pigment extracts could contribute to the observed higher levels of antioxidant activity of outer fractions of durum wheat compared to antioxidant activity of acidified methanol extracts.

### 3.4. Relationship between, Total Phenolic Compounds, Yellow Pigments and Antioxidant Activity

Phenolic compounds function as free radical scavengers and form part of the antioxidant system by donating hydrogen or electrons depending on their chemical structure [31]. The Pearson correlation coefficient between TPC and ABTS radical scavenging activity of whole meal and milling factions is very strong (R = 0.996 and 0.995 for Navigator and Strongfield, respectively, *p* < 0.05), indicating the significant contribution of phenolics to the overall antioxidant capacity of the durum wheat. The strong correlation between total phenolics and antioxidant activity in wheat has been reported by several authors [10,27,32]. There was no relationship between TYP content and the antioxidant activity of water-saturated butanol extracts of whole meal and milling fractions of both cultivars examined in this study. The antioxidant capacity of yellow pigments was probably masked by the strong antioxidant activity of phenolic compounds. However, Ndolo and Beta (2013) reported significant correlations between carotenoid content and DPPH scavenging activity in both whole grain and endosperm only fraction [9]. Yellow pigments in debranned flour were also found to be strongly correlated with ABTS scavenging activity, although such correlation was insignificant in bran [10].

### 3.5. Separation of Yellow Pigments from Durum Wheat Milling Fraction Using UPLC

**Figure 3:**
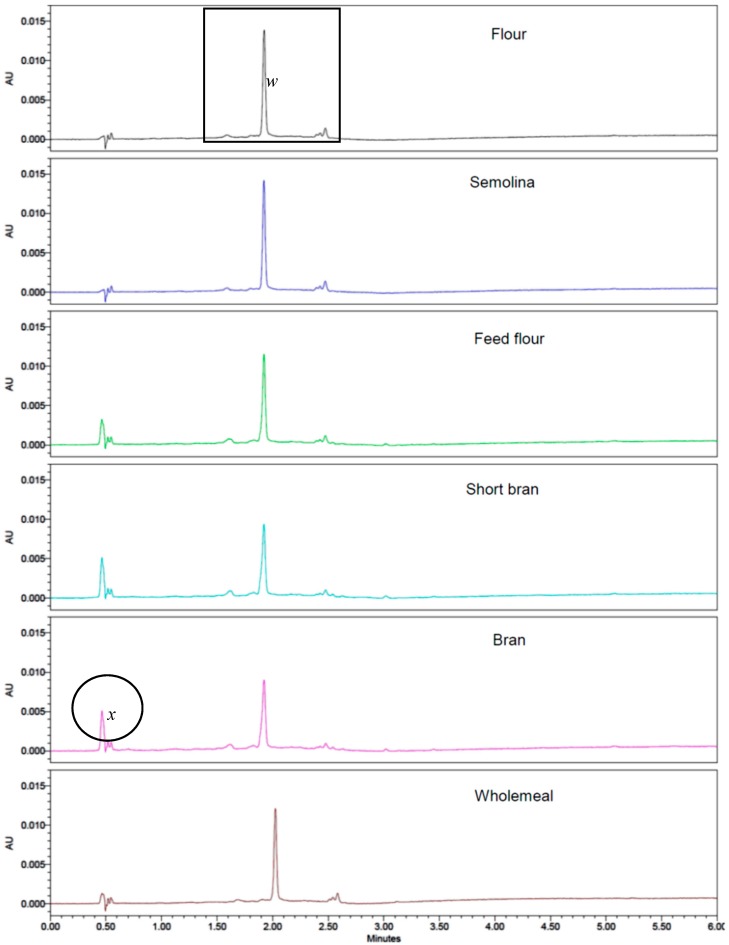
Ultra-performance liquid chromatography (UPLC) chromatograms of flour, semolina, feed flour, short bran, bran, and whole wheat; *x* = unknown compound; *w =* lutein and lutein isomers.

Pigment separation and identification were based on the method of Hung and Hatcher (2011) that separated carotenoids in durum wheat using the same UPLC equipment and column as in this study [23]. Carotenoid identification was accomplished by comparing UV-Visible absorption spectra in literature and the elution sequence according to Hung and Hatcher (2011) and Ndolo and Beta (2013) [9,23]. Carotenoids were quantified based on their absorption at 449 nm. Peaks in zone *w* (Figure 3) were considered carotenoids according to their absorption maxima. Lutein was the dominant carotenoid in durum wheat as reported in the literature [12,26]. Other components in zone *w* exhibited typical carotenoid spectra associated with 13-cis lutein and 9-cis lutein isomers [9,13]. Lutein increased gradually from representing 75% of total carotenoids in bran fractions to 83% in semolina (Table 2) when only peaks absorbing optimally at 449 nm were taken into account. The minor components of lutein isomers contributed between 15.3–20.0% of total carotenoids (Table 2).

**Figure 4:**
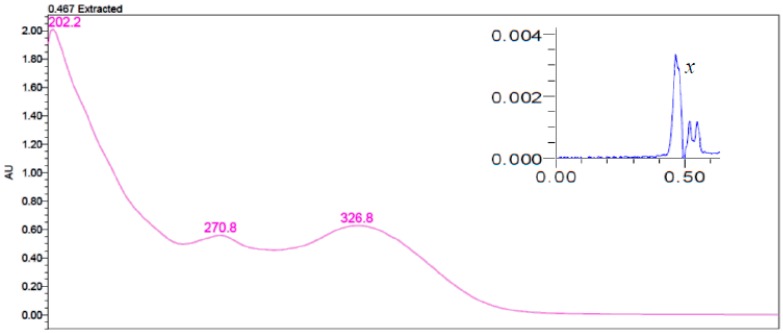
Absorption spectrum of peak *x* eluted at retention time (Rt) 0.47 in bran, short bran, and flour feed fractions.

Peak *x* (Figure 3) with retention time of 0.45 min was detected in bran, short bran, and feed flour fractions. However, this peak did not exhibit the same carotenoid spectra as those in zone *w* even though it was detected at 449 nm. Peak *x* displayed UV absorption spectra (Figure 4) that can be associated with phenolic compounds [33]. The intensity of the peak *x* was lower in whole meal than in bran, short bran, and feed flour fractions due to the dilution of the endosperm. Since phenolic acids absorb in the UV region of the spectrum, compounds with phenolic type absorption spectra are not expected to absorb at the same wavelength as carotenoids. While peak *x* showed high absorbance at 280 nm, which is the optimum wavelength for phenolic acids, no carotenoid peak was detected at this wavelength. The presence of peak *x* in grain outer layers but absent in endosperm fractions also support our assumption that these compounds are phenolic in nature. The identity of the phenolic compounds could not be ascertained as it is likely that only the absorbance tails of the phenolics extended into the visible region of the spectrum. Mazzoncini et al. (2015) determined the phenolic composition of bran and refined white flour for winter wheat cv. *Bologna* [34]. Of the eight phenolic acids identified, *p*-coumaric acid and caffeic acid were present in bran but were not detected in white flour. Identification or even removal of peak *x* may be required in order to quantify carotenoids in durum wheat accurately. Levizou et al. (2004) suggested the use of polyvinylpolypyrrolidone or polythyleneglycol to precipitate phenolic compounds before the measurement for carotenoids [11].

If peak *x* is excluded for quantification, lutein represents 75–82% of the total carotenoids for all milling fractions (Table 2). For fractions from the grain outer layers, peak *x* is 18–26% of total carotenoids if it is included for calculation, and the contribution of lutein decreased to 50–60% of total carotenoids (Table 3). It would have appeared that lutein content of the fractions from grain outer layers is significantly lower than that in semolina and flour. The sum of peak *x* and lutein was similar in bran, short bran, and feed flour (Table 3) as was the content of lutein only without peak *x* (Table 2). In light of these observations, chromatographic methods would be preferred to spectrophotometry to identify and quantify carotenoids accurately.

## 4. Conclusions

The TYP distribution pattern in durum wheat milling fractions varies with genotypes. Yellow pigment concentration can increase or decrease from the outer layers to the endosperm of durum wheat kernel. Phenolic and yellow pigment antioxidants are unevenly distributed in various durum wheat milling fractions. Phenolic and yellow pigment extracts from outer grain layers exhibit higher levels of antioxidant activity compared to semolina and flour fractions. Both TPC and TYP correlate strongly with antioxidant activity. In addition to carotenoids, the co-extracted phenolic compounds in the yellow pigment extracts of outer fractions could contribute to the higher level of antioxidant activity than that in the phenolic extracts. There was an unknown peak during carotenoid separation with UPLC, which exclusively existed in the fractions from outer layers of durum wheat. Based on its UV absorption spectrum, this peak could be composed of phenolic type compounds co-extracted with carotenoids. These co-extracted phenolic compounds could result in significant carotenoid overestimation when non-specific colorimetric methods are used to estimate carotenoid content. To the best of our knowledge, this is the first study to separate these “unknown pigments” reported to interfere with carotenoid quantification in durum wheat. Further study is needed to fully characterize these compounds co-extracted with carotenoids.

## Figures and Tables

**Table 1:** Total yellow pigment (TYP) content, total phenolic content (TPC) of methanol, and yellow pigment extracts of milling fractions.

Fraction	TYP (μg/g)		TPC Methanol Extract (mg CE/100 g ^b^)		TPC Yellow Pigment Extract (mg CE/100 g ^b^)	
	Navigator	Strongfield	Navigator	Strongfield	Navigator	Strongfield
Flour	8.55 a ± 0.13 ^a^	7.83 d ± 0.01	33.8 c ± 5.0 ^b^	36.1 c ± 7.2	22.6 c ± 2.7	20.1 d ± 3.0
Semolina	8.65 a ± 0.09	7.91 cd ± 0.06	32.1 c ± 5.6	33.1 c ± 6.6	17.3 c ± 0.7	17.4 d ± 1.5
Feed flour	8.07 b ± 0.09	8.64 b ± 0.02	77.1 b ± 3.9	88.1 b ± 2.3	53.2 a ± 5.0	55.5 b ± 2.9
Short bran	7.40 c ± 0.00	8.94 a ± 0.09	101.2 a ± 10.4	116.1 a ± 3.2	67.3 a ± 6.8	73.3 a ± 2.8
Bran	7.42 c ± 0.15	8.90 a ± 0.02	92.8 ab ± 1.7	103.4 ab ± 6.3	62.1 a ± 4.9	68.4 a ± 6.8
Wholewheat	8.30 ab ± 0.18	8.04 c ± 0.05	45.7 c ± 2.1	46.8 c ± 1.9	35.5 b ± 3.5	35.3 c ± 1.9

^a^ Values are mean ± standard deviation. Values with different letters in each column are statistically different at *p* < 0.05; ^b^ mg catechin equivalents/100 g.

**Table 2:** Percentage composition of lutein in the durum wheat milling fractions excluding the unknown compounds.

Fraction	Navigator		Strongfield	
	Lutein	Lutein Isomers	Lutein	Lutein Isomers
Flour	81.1 ± 1.7	15.4 ± 1.7	81.4 ± 1.5	16.6 ± 0.8
Semolina	80.9 ± 1.3	15.4 ± 0.8	82.6 ± 0.9	16.5 ± 0.6
Feed flour	79.4 ± 1.1	18.8 ± 1.6	76.5 ± 1.0	19.0 ± 0.8
Short bran	79.2 ±1.7	18.5 ± 1.6	75.4 ± 1.0	20.0 ± 1.3
Bran	80.2 ± 2.3	18.5 ± 1.6	76.5 ± 0.8	18.7 ± 0.9
Wholewheat	82.0 ± 1.7	16.0 ± 0.8	81.5 ± 2.1	15.3 ± 1.4

**Table 3:** Percentage composition of lutein including unknown compounds in the bran, short bran, and feed flour fractions.

Fraction	Navigator			Strongfield		
	Unknown (*x*)	Lutein	Lutein + *x*	Unknown (*x*)	Lutein	Lutein + *x*
Feed flour	18.3 ± 3.7	59.5 ± 3.7	77.8 ± 2.8	19.5 ± 2.8	55.8 ± 3.3	75.3 ± 2.0
Short bran	23.3 ± 2.5	54.5 ± 4.4	77.8 ± 2.8	26.0 ± 1.2	50.3 ± 2.1	76.2 ± 1.2
Bran	23.2 ± 1.8	55.2 ± 2.7	78.4 ± 1.9	25.7 ± 1.8	51.2 ± 2.0	76.9 ± 1.1

## Abbreviations

ABTS, 2,2′-azinobis (3-ethylbenzothiazoline-6-sulfonic acid); CE, catechin equivalents; CWAD, Canada western amber durum; CWRS, Canada western red spring; DPPH, 2,2-diphenyl-1-picrylhydrazyl; Rt, retention time; TE, Trolox equivalents; TPC, total phenolic content; TYP, total yellow pigment; UPLC, ultra-performance liquid chromatography; UV; ultra violet; WSB, water-saturated butanol.
